# Metabolomic Analysis of *Morus* Cultivar Root Extracts and Their Ameliorative Effect on Testosterone-Induced Prostate Enlargement in Sprague-Dawley Rats

**DOI:** 10.3390/ijms21041435

**Published:** 2020-02-20

**Authors:** Young-Jin Choi, Jae In Lee, Meiqi Fan, Yujiao Tang, Eun-Jung Yoon, Young Bae Ryu, Eun-Kyung Kim

**Affiliations:** 1 Division of Food Bioscience, College of Biomedical and Health Sciences, Konkuk University, Chungju 27478, Korea; choijang11@kku.ac.kr (Y.-J.C.); fanmeiqi@kku.ac.kr (M.F.); yuanxi00@126.com (Y.T.); 2 Natural Product Material Research Center, Korea Research Institute of Bioscience and Biotechnology, Jeongeup 56212, Korea; Lji613@kribb.re.kr; 3 School of Bio-science and Food Engineering, Changchun University of Science and Technology, Changchun 130600, China; 4 Department of Physical Education, Korean National University of Education, Cheongju 28173, Korea; 315836@naver.com

**Keywords:** *Morus* root, UPLC–QTOF-MS, benign prostatic hyperplasia, testosterone-induced prostate enlargement model

## Abstract

We investigated the metabolite changes of *Morus* roots (MRs) according to different cultivar families (*Simheung*, *Daesim*, *Cheong-il*, *Sangchon*, *Daeseong*, *Suhong*, *Suwon*, and *Igsu*) using ultra-performance liquid chromatography–quadrupole time-of-flight mass spectrometry (UPLC–QTOF-MS) to understand the relationship between different cultivars and metabolite changes. Data were analyzed by partial least squares discriminant analysis (PLS-DA), and samples were successfully separated in PLS-DA scores. Eight metabolites in the electrospray ionization (ESI)-positive mode and 16 metabolites in the ESI-negative mode contributed to the separation in PLS-DA. Our data suggest that comparative analysis of MR metabolites according to different cultivars is useful to better understand the relationship between the different cultivars and metabolite changes. Furthermore, we analyzed the MRs for their ability to improve benign prostatic hyperplasia (BPH). LNCaP cells were used to evaluate the prostate-specific antigen (PSA) inhibitory activity of MRs, and, amongst them, the extract with the highest activity was selected. *Igsu* demonstrated the highest inhibition effect of prostate-specific antigen (PSA) expression among the MR cultivars. *Igsu* was also evaluated by administration in a testosterone-induced benign prostatic hyperplasia model in Sprague-Dawley rats. *Igsu* was shown to ameliorate BPH as evidenced by the prostate index, expression of androgen receptor (AR) signaling-related protein, growth factors, cell proliferation-related proteins, apoptosis-related proteins, mitogen-activated protein kinase (MAPK) signaling proteins, and histological analysis. Hence, this study strongly suggests that *Igsu* may have a beneficial effect of on BPH.

## 1. Introduction

The prostate produces 30% of the semen volume. The prostate fluid, secreted by the prostate gland, provides nutrition for the spermatic cells, protects them, and helps them live longer and be more mobile. The prostate gland is in contact with the bladder in the upper part and is fixed by the urogenital septum in the lower part [1]. As life expectancy increases worldwide, the prevalence of various age-related diseases continue to rise. In men, benign prostatic hyperplasia (BPH) is a common age-related disease. BPH refers to the proliferation of smooth muscle and epithelial cells located in the transition zone of the prostate, and it leads to morbidity due to urinary symptoms [2]. BPH is also associated with lower urinary tract symptoms (LUTS) including urinary intermittency, frequency, straining, urgency, weak stream, and incomplete emptying [3,4,5,6]. LUTS affects the daily activities of affected men, reducing their quality of life.

The exact mechanism of BPH is not known, and several complex factors such as aging, diet, environment, heredity, inflammation, sex hormone imbalance, growth factors, and apoptosis and proliferation of the prostate cells are believed to affect the development of BPH [7]. The prostate gland is an androgen-dependent male organ. In the prostate, the major androgens such as testosterone and dihydrotestosterone (DHT), produced via the conversion of testosterone by 5-alpha-reductase-type 2 (5AR2), play a crucial role in the growth, proliferation, and maintenance of the prostate. Metabolic maintenance of the prostate is regulated by gene expression, mediated by the binding of DHT and androgen receptor (AR). However, a reduction in testosterone due to aging increases the expression of 5AR2, which converts testosterone to DHT, as well as the expression of AR, which binds to DHT, to maintain constant levels of DHT in prostate. Gene expression, due to the combination of AR and DHT, increases the expression of prostate-specific antigen (PSA) and growth factors such as epidermal growth factor (EGF), vascular endothelial growth factor (VEGF), and insulin-like growth factor-1 (IGF-1), which contribute to the development of BPH. In addition, it is also known that transforming growth factor (TGF)-β1 is overexpressed in BPH. On the other hand, excessive expression of growth factors affects MAPK-signaling. MAPK signal transduction is deeply involved in cell inflammation, environmental stress, cell proliferation, and apoptosis, which also affect BPH [8].

Among pharmacological therapies for BPH, finasteride (Fi) and dutasteride are effective synthetic agents. However, these agents have adverse side effects such as erectile dysfunction, diminished sexual drive, and reduced semen volume during ejaculation [9,10]. Therefore, those desiring fewer side effects and effective treatments are interested in alternative medicines made from natural substances [11].

*Morus*, also called the mulberry plant, is a monoecious or dioecious plant, growing 10–12 m in height, with worldwide distribution; the majority of species are found growing in the Asian and Indo-Pacific islands. There are 24 species of *Morus* and one subspecies, with at least 100 known varieties. The leaves are the sole food for silkworms, and the fruits are edible and healthy for humans [12,13]. Many studies reported that the roots, leaves, and branches of *Morus* show significant effects in chronic diseases. For instance, the segregated compounds identified from *Sang-Bai-Pi*, the root bark of *Morus alba* L., were isoprenylated flavonoids such as sanggenon A, sanggenon N, and sanggenon P [14], condensation compounds of Diels–Alder type adducts such as kuwanon A and mulberrofuran G [15], triterpenoids such as ursolic acid, and benzofurans such as moracin O and moracin M [16]. These compounds were indicated to have beneficial effects such as a whitening effect, in addition to anti-oxidant [17,18], anti-inflammatory [19], and adaptogenic activity [20].

In this study, we investigated the metabolite changes of *Morus* root (MR) according to different cultivars (*Simheung*, *Daesim*, *Cheong-il*, *Sangchon*, *Daeseong*, *Suhong*, *Suwon*, and *Igsu*) using ultra-performance liquid chromatography–quadrupole time-of-flight mass spectrometry (UPLC–QTOF MS). Furthermore, this study also investigated the effect of *Igsu* extract on BPH in a rat model for testosterone propionate (TP)-induced BPH by selecting the roots with the most effective inhibition of PSA expression in the LNCaP cell line.

## 2. Results

### 2.1. Metabolomic Analysis of MRs According to Different Cultivars

**Figure 1:**
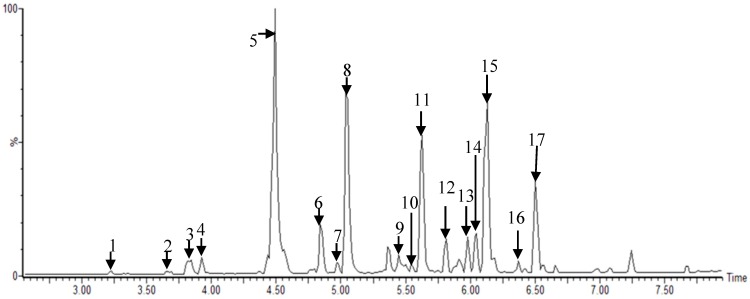
Representative ultra-performance liquid chromatography–quadrupole-time-of-flight mass spectrometry (UPLC–Q-TOF MS) profiles of *Morus* root cultivars: 1, kuwanon X/Y/P; 2, mulberrofuran J/C; 3, kuwanon L; 4, mulberrofuran I; 5, kuwanon K/G; 6, sanggenon N/I; 7, kuwanon O; 8, kuwanon H/N; 9, sanggenol M; 10, kuwanon A/B; 11, kuwanon E; 12, kuwanon M; 13, kuwanon M/mulberrofuran U; 14, kuwanon D/F/T; 15, morusin; 16, kuwanon A/B, and 17, kuwanon U analyzed in electrospray ionization (ESI)-negative mode.

**Figure 2:**
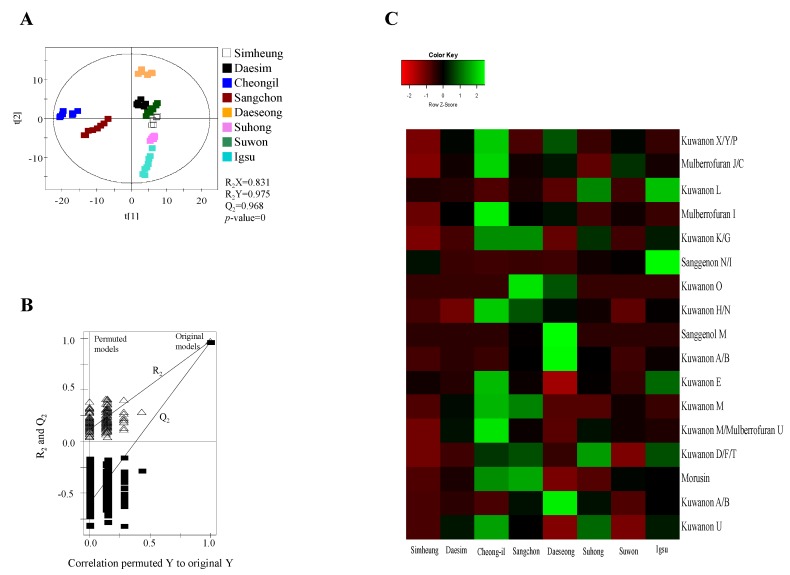
Partial least squares discriminant analysis (PLS-DA) scores (**A**), their quality parameters (**B**), and heat map (**C**) for ESI-negative mode from *Morus* roots with different cultivars (*Simheung*, *Daesim*, *Cheong-il*, *Sangchon*, *Daeseong*, *Suhong*, *Suwon*, and *Igsu*). The quality of the PLS-DA score plots was evaluated by R_2_X, R_2_Y, Q_2_, and *p*-values (**A**) and validated by permutation tests (**B**). The heat map was drawn by R with ggplot2, and the green–red color represents the *z*-score transformed raw data of *Morus* root metabolites with significant differences among sample groups. Red and green colors indicate a decrease and an increase in metabolite level, respectively.

Metabolites were extracted using 80% methanol from MR according to different cultivars (*Simheung*, *Daesim*, *Cheong-il*, *Sangchon*, *Daeseong*, *Suhong*, *Suwon*, and *Igsu*), which were analyzed by UPLC–QTOF MS (Figure 1 and Figure S1, Supplementary Materials). The results were statically analyzed by partial least squares discriminant analysis (PLS-DA) (Figure 2 and Figure S2, Supplementary Materials). The PLS-DA model quality parameters (R_2_X = 0.845, R_2_Y = 0.967, and Q_2_ = 0.952 for positive mode; R_2_X = 0.831, R_2_Y = 0.975, and Q_2_ = 0.968 for negative mode) for fitness and predictability (Figure 2A and Figure S2B, Supplementary Materials), and the values obtained from cross-validation by a permutation test (R_2_ intercept < 0.4, Q_2_ intercept < −0.2, and *p*-value = 0 for electrospray ionization (ESI)-positive mode; R_2_ intercept < 0.4, Q_2_ intercept < −0.2, and *p*-value = 0 for ESI-negative mode) indicated that the PLS-DA plots were statistically acceptable (Figure 2A and Figure S2A,B, Supplementary Materials).

A total of 178 metabolites in ESI-positive mode and 279 metabolites in ESI-negative mode were detected in the 80% methanol extract of MR using UPLC–QTOF MS. Among these metabolites, statistical analysis of the normalized metabolites using ANOVA with Duncan’s test (*p* < 0.05) showed that 159 metabolites analyzed by ESI-positive mode and 270 metabolites analyzed by ESI-negative mode significantly changed with different cultivars. Of these metabolites, eight metabolites in ESI-positive mode including mulberrofuran G, mulberrofuran I, kuwanon G, kuwanon D/F/T, kuwanon H, luteolin-methyl ester-glycoside fragment, kuwanon A/B, and morusin, and 16 metabolites in ESI-negative mode including kuwanon X/Y/P, mulberrofuran J/C, kuwanon L, mulberrofuran I, kuwanon K/G, sanggenon N/I, kuwanon O, kuwanon H/N, sanggenol M, kuwanon A/B, kuwanon E, kuwanon M, mulberrofuran U, kuwanon D/F/T, morusin, and kuwanon U (Table 1) with variable importance in the projection (VIP) > 0.7 were identified as metabolites contributing to the separation among the sample groups on the PLS-DA score plots.

**Figure 3:**
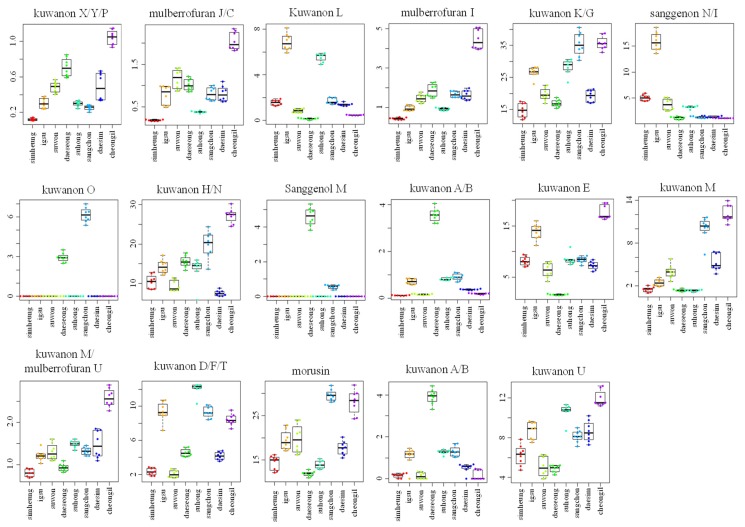
Box plot graphs of *Morus* roots with different cultivars (*Simheung*, *Igsu*, *Suwon*, *Daeseong*, *Suhong*, *Sangchon*, *Daesim*, and *Cheong-il*) for ESI-negative mode. Box plot graphs show the minimum, first quartile, median, third quartile, maximum, and outliers.

For visualizing the changes in metabolite levels according to different cultivars, a heat map prepared using *z*-score transformed raw data of *Morus* metabolites and a box plot were plotted (Figure 2C and Figure 3 and Figures S2C and S3, Supplementary Materials). In ESI-positive mode, the relative intensity levels of mulberrofuran G, mulberrofuran I, kuwanon H, and luteolin-methyl ester glycoside fragment in *Cheong-il* cultivar were lower than in the other cultivars. The intensity level of kuwanon G in *Cheong-il* and *Sangchon* cultivars was the same and higher than in the other cultivars. The intensity level of kuwanon D/F/T in *Igsu* and *Suhong* cultivars was higher than in the other cultivars. The intensity levels of kuwanon A/B and morusin were highest in the *Sangchon* cultivar. In ESI-negative mode, the intensity levels of kuwanon X/Y/P, mulberrofuran J/C, mulberrofuran I, kuwanon K/G, kuwanon H/N, kuwanon E, kuwanon M, mulberrofuran U, and kuwanon U were highest in the *Cheong-il* cultivar. The intensity levels of kuwanon L and sanggenon N/I in *Igsu* cultivar were higher than in other cultivars. The intensity level of kuwanon O in the *Sangchon* cultivar and that of sanggenol M and kuwanon A/B in the *Daeseong* cultivar were higher than that in the other cultivars.

### 2.2. Effect of Igsu Administration on Prostate Tissue Weight in Rats

In this study, we analyzed the metabolites of MR according to different varieties and selected eight cultivars that contained the most active compounds. The eight varieties were administered to LNCaP cells with DHT to evaluate the inhibition of PSA expression (Figure S4, Supplementary Materials). *Igsu*, which demonstrated the highest inhibition effect of PSA expression among the MR cultivars, was administered to the TP-induced BPH rat model to evaluate its ameliorating effect on BPH. Before the animal experiment, we demonstrated safety of the eight varieties via cell viability assay (Figure S5, Supplementary Materials). Moreover, *Igsu* was determined anti-benign prostatic hyperplasia in vitro system (Figures S6 and S7, Supplementary Materials).

**Figure 4:**
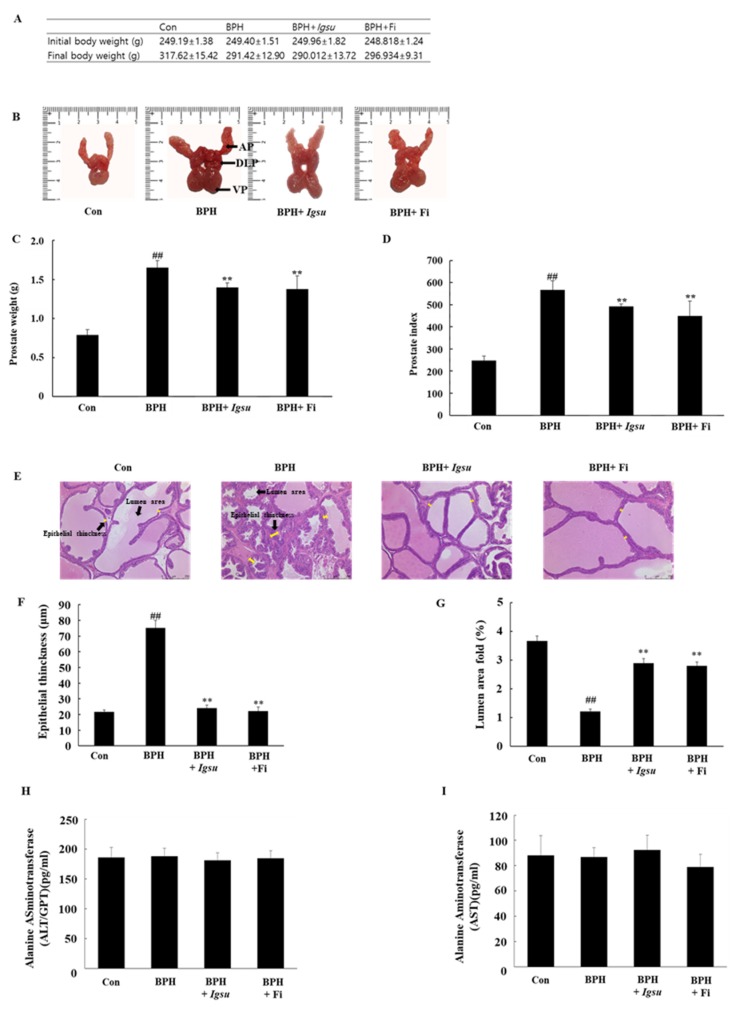
Evaluation of prostate weight and toxicity according to administration of *Igsu* in benign prostatic hyperplasia (BPH). (**A**) Body weight of the rats in the different groups. (**B**) Dorsolateral prostate lobe (DLP), anterior prostate lobe (AP), and ventral prostate lobe (VP) dissected from the different groups. (**C**) The total prostate weight of the rats. (**D**) Prostate indexes in different groups. (**E**) Hematoxylin and eosin (H&E) staining of prostate tissue sections. (**F**) Epithelial thickness of the prostate tissues. (**G**) Lumen area fold in the prostate tissues. (**H**) Concentration of alanine transaminase (ALT) in the serum. (**I**) Concentration of aspartate transaminase (AST) in the serum. Con, normal control group; BPH, testosterone propionate (TP) (3 mg/kg/day)/corn oil injection; BPH + *Igsu*, *Igsu*-treated BPH group; BPH + Fi, finasteride (Fi)-treated BPH group. Data are presented as the mean ± standard error of the mean (SEM) (*n* = 8). Significant differences at ^##^ *p* < 0.01 compared with the Con group. Significant differences at ** *p* < 0.01 compared with the BPH group.

The changes in body weight, images of the excised prostates (dorsolateral prostate lobe (DLP), anterior prostate lobe (AP), and ventral prostate lobe (VP)), total prostate weight, and prostate index (prostate weight in g per 100 g body weight) in the control and experimental groups are shown in Figure 4A–D, respectively. The body weight of the BPH-induced group was lower than that of the control group, although the difference was not significant. The prostate weight of the BPH group (567.43 ± 39.67, *p* < 0.01) was significantly higher than that of the control group (247.82 ± 20.01). However, the prostate weight of the BPH + *Igsu* group (507.73 ± 32.05, *p* < 0.01) was significantly reduced compared to that of the BPH group. The prostate weight of the BPH + Fi group (464.07 ± 66.38, *p* < 0.01) was also decreased markedly compared to the BPH group. The prostate index was also significantly decreased in the BPH + *Igsu* and BPH + Fi groups compared to the BPH group, consistent with decrease in the prostate weights. The prostate index results were similar for the group administered *Igsu* and that administered Fi. In addition, *Igsu* was well tolerated as the treated animals did not show any signs of toxicity (Figure 4H,I).

### 2.3. Effects of Igsu Administration on Prostate Histology in Rats

We measured epithelial thinning and lumen area in the prostate tissue by hematoxylin and eosin (H&E) staining. Histological analysis of the prostate tissue revealed that the control group had normal cell morphology; however, the thickness of the prostate epithelium was significantly increased in the BPH group compared to that in the control group. The proximal part of the prostate in the BPH group was significantly lower than in the control group. In the *Igsu-* and Fi-treated groups, the thickness of the epithelial cells was reduced to the level of the control group. Furthermore, the luminal area was also significantly increased compared to that in the BPH group (Figure 4E–G).

### 2.4. Effect of Igsu Administration on the Levels of DHT in Prostate Tissue of Rats

**Figure 5:**
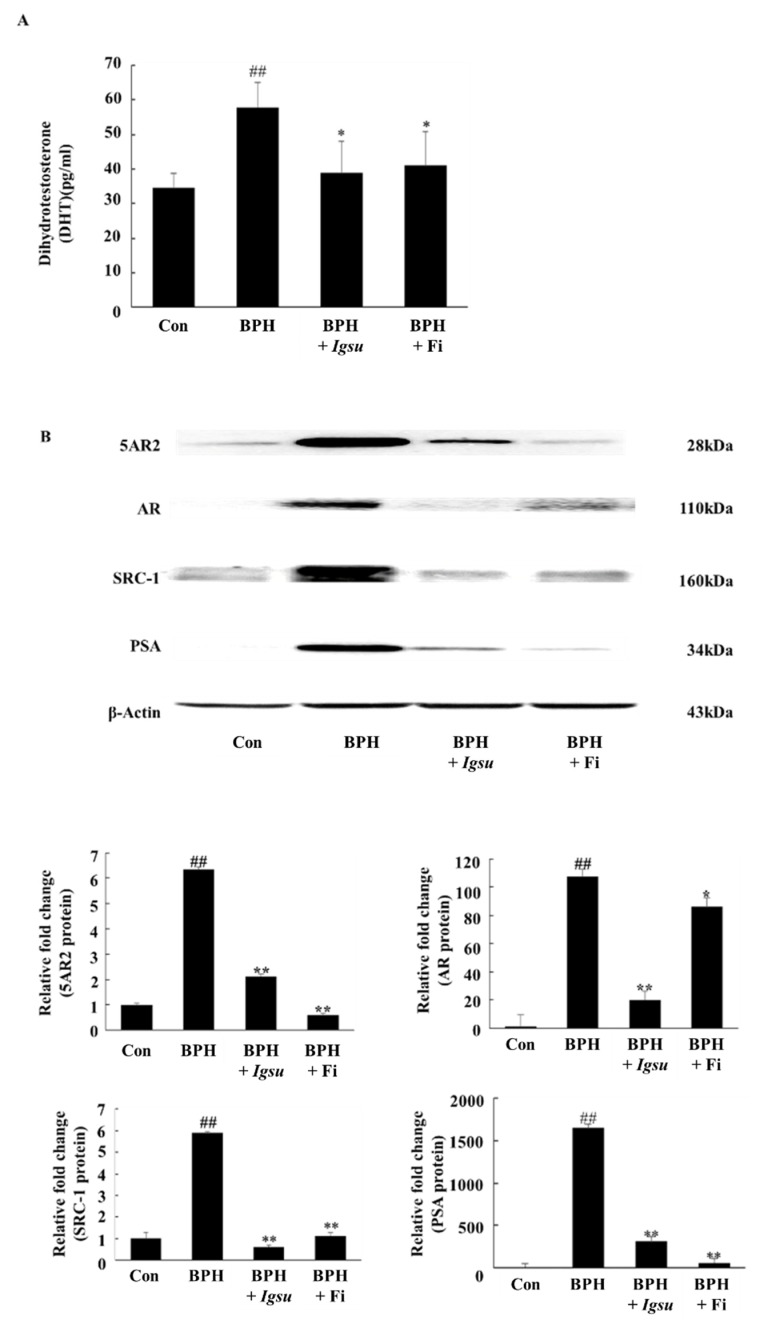
Expression of androgen receptor (AR) signaling-related factors in prostate tissue following administration of *Morus* root (MR) in BPH. (**A**) Concentration of dihydrotestosterone (DHT) in the prostate tissues. (**B**) Expression of 5-alpha-reductase-type 2 (5AR2), AR, steroid receptor coactivator (SRC)-1, and prostate-specific antigen (PSA) proteins in prostate tissues. Con, normal control group; BPH, TP (3 mg/kg/day)/corn oil injection; BPH + *Igsu*, *Igsu*-treated BPH group; BPH + Fi, Fi-treated BPH group. Data are presented as the mean ± SEM (*n* = 8). Significant differences at ^##^ *p* < 0.01 compared with the Con group. Significant differences at * *p* < 0.05, ** *p* < 0.01 compared with the BPH group.

As shown in Figure 5A, the DHT level in the prostate of rats in the TP-induced BPH group (57.84 ± 7.33 pg/mL serum, *p* < 0.05) was markedly higher than in rats in the control group (34.57 ± 4.16 pg/mL). However, prostatic DHT levels in rats in the Fi-treated group (41.09 ± 9.88 pg/mL, *p* < 0.05) and the *Igsu* R-treated group (38.99 ± 9.32 pg/mL, *p* < 0.05) were significantly lower than in the BPH group). We confirmed that *Igsu* effectively inhibited the expression of 5AR2, thereby inhibiting DHT production (Figure 5B).

### 2.5. Effect of Igsu on Protein Expressions of 5AR2, AR, SRC-1, and PSA in Prostate Tissues of Rats

Figure 5B shows the expression of 5AR-2, AR, steroid receptor coactivator (SRC)-1, and PSA by Western blotting analysis. The protein levels of 5AR-2, AR, SRC1, and PSA were increased in the BPH group (*p* < 0.01) compared to those in the control group. However, the expressions of 5AR-2, AR, SRC-1, and PSA proteins were significantly downregulated following administration of *Igsu* compared to the BPH group. Specifically, AR and SRC-1 were more inhibited in the *Igsu* group than in the Fi treatment group. In the above results, *Igsu* showed an inhibitory effect on anti-5AR2. This resulted in the conversion of testosterone to DHT, which inhibited the binding of AR and SRC-1, which reduced the expression of PSA. These data show that the *Igsu* treatment effects are similar to those of Fi treatment and, therefore, *Igsu* can potentially be used as a natural treatment for BPH.

### 2.6. Effect of Igsu Administration on Expression of Growth Factor-Related Protein in Prostate Tissue of Rats

**Figure 6:**
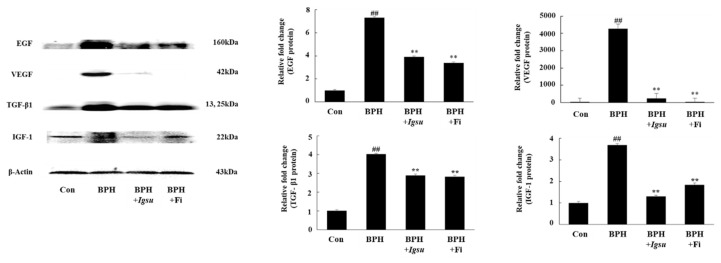
Expression of growth factors in prostate tissue following administration of *Igsu* in BPH. Expression of epidermal growth factor (EGF), vascular endothelial growth factor (VEGF), transforming growth factor (TGF)-β1, and insulin-like growth factor (IGF)-1 proteins in prostate tissues. β-actin served as a loading control. Con, normal control group; BPH, TP (3 mg/kg/day)/corn oil injection; BPH + *Igsu*, *Igsu*-treated BPH group; BPH + Fi, Fi-treated BPH group. Data are presented as the mean ± SEM (*n* = 8). Significant differences at ^##^ *p* < 0.01 compared with the Con group. Significant differences at ** *p* < 0.01 compared with the BPH group.

We measured the protein levels of EGF, VEGF, TGF-β1, and IGF-1 in prostate tissue by Western blotting. The protein levels of EGF, VEGF, TGF-β1, and IGF-1 in the BPH group (*p* < 0.01) were significantly increased compared to those in the control group. However, both Fi and MR treatment significantly reduced the level of these proteins compared to the BPH group (Figure 6).

### 2.7. Effect of Igsu on Phosphorylation of MAPK Signaling in Prostate Tissue of Rats

**Figure 7:**
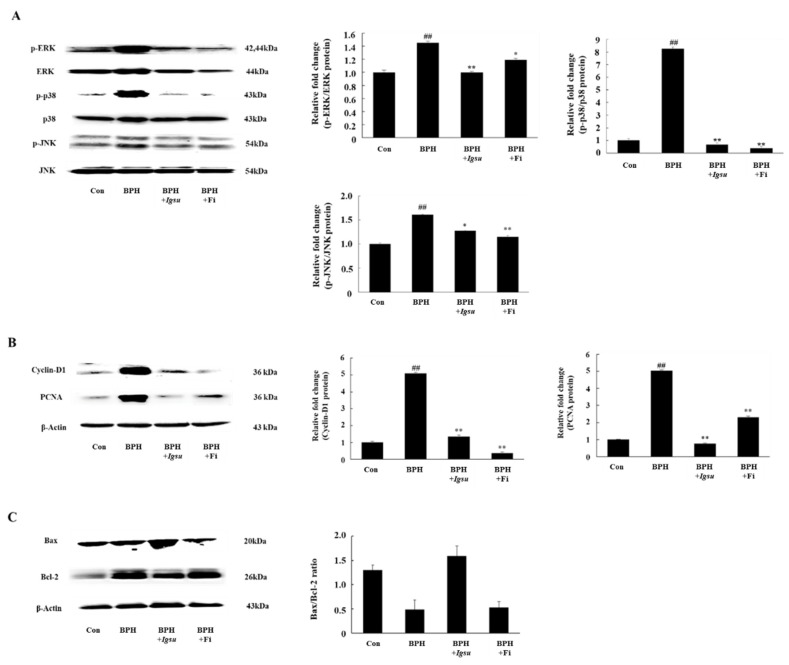
Expression of mitogen-activated protein kinase (MAPK)-, cell proliferation-, and apoptosis-related factors in prostate tissue following administration of *Igsu* in BPH. (**A**) Expression of MAPK signaling pathway mediators in prostate tissue. (**B**) Expression of cell proliferation-related proteins, proliferating cell nuclear antigen (PCNA) and cyclin D1, in prostate tissues. (**C**) Expressions of apoptosis-related proteins and B-cell lymphoma 2 (Bcl-2) X-associated (Bax)/Bcl-2 ratio in prostate tissues. Con, normal control group; BPH, TP (3 mg/kg/day)/corn oil injection; BPH + *Igsu*, *Igsu*-treated BPH group; BPH + Fi, Fi-treated BPH group. Data are presented as the mean ± SEM (*n* = 8). Significant differences at ^##^ *p* < 0.01 compared with the Con group. Significant differences at * *p* < 0.05, ** *p* < 0.01 compared with the BPH group.

In this study, we measured expression of MAPK protein in the prostate. We investigated the phosphorylation status of extracellular signal-related kinase (ERK), c-Jun N-terminal kinase (JNK), and p38 in prostate tissue. As shown in Figure 7A, the phosphorylation of MAPK was significantly increased in the BPH group compared to the control group (*p* < 0.01). On the other hand, the phosphorylation of ERK (*p* < 0.01), JNK (*p* < 0.05), and p38 (*p* < 0.01) in the BPH + *Igsu* group showed a significant reduction compared to the BPH group.

### 2.8. Effect of Igsu Administration on Prostate Proliferation-Related Protein Expression in Prostate Tissue of Rats

We found that the protein levels of PCNA and cyclin D1 were significantly increased in the BPH group compared to the control group. However, Fi or *Igsu* treatment significantly reduced the expression of proliferating cell nuclear antigen (PCNA) and cyclin D1 compared to the BPH group (Figure 7B). These results suggest that *Igsu* treatment inhibited excessive cell proliferation and returned the expression of PCNA and cyclin D to normal levels.

### 2.9. Effect of Igsu Administration on Expression of Apoptosis Related Proteins in Prostate Tissue of Rats

We found that the B-cell lymphoma 2 (Bcl-2)/Bcl-2 X-associated (Bax) ratio was significantly higher in the BPH group than in the control group, while it was significantly lower in the *Igsu* group than in the BPH group. The ratio was also lower in the Fi group than in the BPH group, although the difference was not statistically significant (Figure 7C).

## 3. Discussion

The prostate is a male hormone-dependent organ. Therefore, male hormones are essential for maintaining the growth, proliferation, and function of the prostate. Testosterone and DHT, especially, play an important role in the prostate cell cycle. In the prostate cells, testosterone is converted by 5AR2 to DHT, an androgen that is five times more active than testosterone [3]. An increase in DHT concentration in prostate tissue is one of the representative clinical markers of BPH. DHT binds to androgen receptor (AR), a member of the nuclear receptor family, and acts biologically as a ligand-reactive transcription factor. In addition, androgen receptor coactivators, such as SRC-1, interact with AR to regulate AR transactivation [21]. As a result, the binding between DHT, AR, and SRC-1 increases the expression of PSA and growth factors in prostate cells. In men with reduced testosterone levels due to aging, the expression of 5AR2 and AR is increased to control endocrine homeostasis [22]. As a result, the binding of DHT and AR increases, which in turn plays a key role in BPH development.

Meanwhile, 5-alpha reductase inhibitors that inhibit the conversion of testosterone to DHT are commonly used to attenuate the symptoms of BPH [23]. Fi is one of the 5-alpha reductase inhibitors currently being used in the treatment of BPH. However, Fi was reported to have some side effects [24]. Therefore, lots of research was actively conducted on BPH from natural plant materials with little side effects. We analyzed the metabolites of MR and found that they contained a large amount of flavonoids (Figure 2 and Figure 3). Based on previous studies showing that flavonoids have an effect on BPH [25], in this study, MRs were measured for their inhibition activity of PSA on LNCaP cells, and *Igsu* showed the highest activity among the eight varieties. Furthermore, the anti-prostate hypertrophy effect of *Igsu* was verified using the TP-induced BPH rat model. The administration of *Igsu* inhibited 5AR2 expression. Consequently, the administration of *Igsu* significantly reduced the level of DHT in prostate tissue compared to the BPH group. Reduced binding of DHT, AR, and SRC-1 resulting from the administration of *Igsu* significantly inhibited PSA expression.

Abnormal AR signaling due to hormonal imbalance increases the expression of growth factors. Growth factors are potent mediators of cell growth, differentiation, and stroma–epithelial interactions in the prostate. Growth factors such as EGF, VEGF, and IGF that are regulated by AR are believed to act as factors that promote prostate growth [26]. Under normal circumstances, TGF-β1 induces apoptosis of prostate cells and offsets mitosis. However, in BPH, TGF-β1 plays a key role in BPH development by contributing to the induction of extracellular matrix proteins and the differentiation of prostatic fibroblasts [27]. In this experiment, *Igsu* significantly inhibited the expression of growth factors such as EGF, VEGF, TGF-β1, and IGF-1 by inhibiting AR signaling-related factors (Figure 6). Reduction of growth factors significantly affected MAPK signaling, cell proliferation, and apoptosis [28].

Decreased expression of growth factors led to downregulation of phosphorylation of the MAPK/ERK pathway signaling mediators. MAPKs represent a group of protein kinases that play an important role in integrating the physiological and pathological stimuli that can affect cell growth, differentiation, and apoptosis [29]. In addition, it was reported that MAPK signaling is activated when stimulated by mitogens such as growth factors. Normal prostate cell growth is regulated by a delicate balance between cell death and cell proliferation. The rates of cell proliferation and cell death are balanced, and continuous cell replacement occurs. However, when cell proliferation activity is increased, or cell death activity is decreased, the balance is upset, causing abnormal prostate growth, leading to BPH. The major protein involved in cell proliferation, PCNA, an acidic nuclear protein, is primarily known as a histological indicator of the gap 1 (G1)/synthesis (S) phase in the cell cycle [30]. Cyclin D1 is the most representative cyclin protein, for which the mechanism of action involves a surveillance gate that regulates cell proliferation [31]. PCNA and cyclin D1 reflect cell proliferation, and increased expression of PCNA and cyclin D1 in the prostate indicates an increase in prostate volume. The balance between apoptosis and cell growth is critical to the normal tissue maintenance of the prostate gland. In apoptosis, stress-activated protein kinase-1/c-Jun N-terminal kinase (SAPK1/JNK) phosphorylates Bax and p38 mitogen-activated protein kinase (p38 MAPK) activates caspase 3, an important factor in cell death [32]. Bcl-2 inhibits the activity of caspase 3 [33]. Therefore, the Bax/Bcl-2 ratio determines cell apoptosis. Abnormal prostatic hyperplasia, such as prostate hypertrophy, can be said to be a response to the imbalance between regulatory factors associated with apoptosis and cell growth. Our results showed that *Igsu* inhibited the phosphorylation of ERK compared to the BPH group. Accordingly, the administration of *Igsu* in BPH decreased the expression of PCNA and cyclin D1, which are major markers of cell proliferation, and it significantly increased the ratio of Bax/Bcl-2, which is a major marker of apoptosis compared to the untreated group. The size of the prostate in the *Igsu*-treated group was also reduced compared to the BPH group. Our results showed that *Igsu* treatment ameliorates BPH comparable to Fi treatment. In previous reported studies, natural plant products such as *Pao pereira*, *Quisqualis indica*, and *Poncirus trifoliata* were shown to attenuate the symptoms of BPH [34,35,36]. The natural plant extracts significantly reduced AR, 5α-reductase, and DHT level in prostate glands of the testosterone-induced BPH rat model. Thereafter, expression of factors related to prostate proliferation such as PCNA and cyclin D1 was also reduced. In addition, the extracts showed an effect of reducing the thickness of the prostate epithelium. The results of the previous studies are very similar to our study, which may be due to the large amounts of flavonoids, polyphenols, and plant sterols found in natural foods. Phytochemicals in natural plants were recently reported to be effective in BPH [25,34,35,36,37].

In our study, the metabolite analysis showed that MR contains a large number of flavonoids and polyphenols. This is the first metabolite analysis of MR, and the data could be used as basic data for MR researches. Furthermore, based on the above results, we suggest the potential use of *Igsu* as a functional food for the prevention and treatment of BPH. Regarding the metabolites of *Igsu*, sanggenon N/I and kuwanon L were the most abundant metabolites of *Igsu*. However, there are currently insufficient studies on the physiological activity of sanggenon N/I and kuwanon L. Therefore, the effect of sanggenon N/I and Kuwanon L of *Igsu* on prostate hyperplasia needs to be elucidated.

## 4. Materials and Methods

### 4.1. Materials

Testosterone propionate (TP) was procured from the Tokyo Chemical Industry Co. (Tokyo, Japan). Protease inhibitor cocktail, finasteride (Fi) (≥97% pure), and dihydrotestosterone (≥99% pure) were purchased from Sigma-Aldrich Inc. (St. Louis, MO, USA). Roswell Park Memorial Institute (RPMI) medium, fetal bovine serum (FBS), and penicillin/streptomycin were purchased from Gibco (Big Cabin, OK, USA).

### 4.2. Sample Preparation

*Morus alba* L. root cultivars (*Simheung*, *Daesim*, *Cheong-il*, *Sangchon*, *Daeseong*, *Suhong*, *Suwon*, and *Igsu*) were cultivated at the Rural Development Administration (Jeonju, Jeonbuk, Korea). The roots were stored at constant room temperature of 25 °C until extraction. *Igsu* was extracted with a bullet blender (Next Advance, NY, USA) using 80% methanol, including terfenadine and zidovudine as internal standards. The supernatant was analyzed using UPLC–QTOF MS (Waters Corporation, Milford. MA, USA).

### 4.3. Ultra-Performance Liquid Chromatography–Quadrupole Time-of-Flight (UPLC-QTOF) MS Analysis

The samples were injected in an Acquity™ UPLC BEH C18 column (2.1 × 100 mm inner diameter, particle size 1.7 μm; Waters) equilibrated with water containing 0.1% formic acid, and eluted in a gradient with acetonitrile (ACN) containing 0.1% formic acid at a flow rate of 0.35 mL/min for 12 (ESI-positive mode) and 16 (ESI-negative mode) min. The column temperature was 40 °C. The eluents were analyzed by QTOF MS in electrospray ionization (ESI)-positive and -negative modes.

In the ESI-positive mode, the voltages of the capillary and sampling cones were 3.0 kV and 40 V, respectively. The desolvation flow rate was 900 L/h, desolvation temperature was 400 °C, and source temperature was set at 100 °C. The TOF MS data were collected in the m/z 100–1500 range with a scan time of 0.2 s. The MS/MS spectra of the extracts were collected in the m/z 50–1500 range by a collision energy ramp from 20 to 45 eV. In the ESI-negative mode, the voltages of the capillary and sampling cones were 2.5 kV and 40 V, respectively. The desolvation flow rate was 800 L/h, desolvation temperature was 300 °C, and source temperature was set at 100 °C. The TOF MS data were collected in the *m*/*z* 50–1500 range with a scan time of 0.2 s. The MS/MS spectra were collected in the *m*/*z* 50–1500 range by a collision energy ramp from 10 to 45 eV.

LockSpray with leucine–enkephalin ([M + H] = 556.2771 Da and [M − H] = 554.2615 Da) was used at a frequency of 10 s in positive and negative mode. Acquisition of the MS data was performed using MassLynx software (Waters), including *m*/*z*, retention time, and ion intensity; all data were extracted with MarkerLynx software (Waters).

### 4.4. Data Processing

Collection, normalization, and alignment of the MS datasets analyzed by UPLC–QTOF MS were obtained using MarkerLynx software (Waters). Peaks were collected using a peak-to-peak baseline noise of 1, noise elimination of 6, peak width at 5% height of 1 s, and an intensity threshold of 10,000. The MS data were aligned with a 0.05-Da mass window and a retention time window of 0.2 min. All mass spectra were normalized with an internal standard ([M + H] = terfenadine and [M − H] = zidovudine). The metabolites were identified using the Human Metabolome Database (HMDB) (www.hmdb.ca), Metabolite and Chemical Entity (METLIN) database (metlin.scripps.edu), Chemspider (www.chemspider.com), literature references, and authentic standards.

### 4.5. Treatment of the LNCaP Cells

LNCaP cells were purchased from the Korean Cell Line Bank (Seoul, Korea, KCLB numbers: 21740). The cells were cultured in RPMI supplemented with 100 mg/mL penicillin/streptomycin, and 10% FBS. They were maintained in a CO_2_ incubator at 37 °C.

The LNCaP cells were seeded onto six-well plates (1 × 10^6^ cells/well) in 2 mL of RPMI medium supplemented with 10% FBS, 100 U/mL penicillin, and 100 mg/mL streptomycin. One day later, the cells were co-incubated with DHT (10 nmol) and *Morus* root cultivars (10 μg/mL) (*Simheung*, *Daesim*, *Cheong-il*, *Sangchon*, *Daeseong*, *Suhong*, *Suwon*, and *Igsu*) for 24 h. In this case, LNCaP cells treated with Fi (1 μg/mL) served as the positive controls. These cells were collected for PCR analysis of PSA expressions.

### 4.6. Analysis of Messenger RNA (mRNA) Expression

For the reverse transcription (RT) polymerase chain reaction (PCR), the total cellular RNA was isolated from the LNCaP cell using TRIzol (Ambion, Austin, TX, USA) according to the manufacturer’s protocol. The first-strand complementary DNA (cDNA) was synthesized using a master mix for cDNA synthesis (Bioneer, Daejeon, Korea). RT-PCR was carried out using a Veriti^®®^ Thermal Cycler (Applied Biosystems, Beverly, MA, USA) according to the manufacturer’s protocol. Briefly, 2 μL of cDNA (100 ng), and 18 μL of dH_2_O with forward and reverse primer solution (1 pM) were mixed to AccuPower^®®^ RT PreMix (Bioneer, Daejeon, Korea). The amplifcation procedure consisted of an initial denaturation step of 5 min at 94 °C, followed by 30 cycles of denaturation at 94 °C for 30 s, annealing at 60 °C for 30 s, and extension at 72 °C for 30 s, followed by a final extension step of 5 min at 72 °C. The primers were purchased from Genotech (Daejeon, Korea) for PSA (forward, 5′–GCT GAC CTG AAA TAC CTG–3′; reverse, 5′–AGC CCC AAG CTT ACC ACC–3′) or GAPDH (forward, 5′–CCA TGG GGA AGG TGA AGG TG–3′; reverse, 5–AAA TGA GCC CCA GCC TTC TC–3′).

### 4.7. Animal Experiments for Testosterone-Induced Benign Prostatic Hyperplasia

#### 4.7.1. Animals

The male Sprague-Dawley (SD) rats (*n* = 32, eight weeks old) with initial body weights of 245–255 g were purchased from Nara Biotech, Co., Ltd. (Pyeongtaek, Korea). The rats were placed in a specific pathogen-free (SPF) room maintained in an air-conditioned (23–25 °C) room and a relative humidity of 50–60% on a 12-h light/dark cycle. Water and a standard laboratory diet were provided ad libitum. All animal care procedures and experiments were approved by the Institutional Animal Care and Use Committee of the Konkuk University (KU17088).

#### 4.7.2. Animal Treatments and Experimental Design

Castration was performed to block off the effects of intrinsic testosterone [38]. BPH was induced in the rats by subcutaneous injection of testosterone following castration. With the exception of the sham operation control group, three groups of rats were anesthetized with intraperitoneal injection of phenobarbital (50 mg/kg) and castrated aseptically to remove bilateral testes. All the castrated rats were subcutaneously injected with TP (3 mg/kg/day) dissolved in corn oil, three days after castration. The BPH model was induced through 28 consecutive days of TP treatment, which was confirmed by histopathological examination of the prostate tissue. One group was served as the control group while the others served as experimental groups. The experimental groups were administrated *Igsu* (100 mg/kg/day) orally for 28 days. Fi, which served as a positive control, was dissolved in distilled water prior to administration (1 mg/kg/day). Following the final injection and overnight fasting, the rats were anesthetized with pentobarbital (50 mg/kg, via intraperitoneal injection). Blood samples were collected, and the prostate tissues were immediately excised and weighed. The prostate index of each rat was calculated as the ratio of the prostate weight to body weight (mg/100 g). The prostate lobe sections were fixed with 10% paraformaldehyde for histological analysis, and the remaining prostate sections were stored at −80 °C for Western blotting analysis.

#### 4.7.3. Hematoxylin and Eosin (H&E) Staining

The prostate tissues were fixed in 10% formaldehyde, dehydrated, and embedded in paraffin. Paraffin was sectioned at 4 μm using a microtome (Leica, Werzlar, Germany). For H&E staining, the sections were stained in hematoxylin for 5 min, and then washed with water for 5 min. Then, the sections were stained in eosin for 30 s, dehydrated, and mounted using routine methods. The slides were examined using the Leica DM6 Research Inverted Phase microscope (Leica, Werzlar, Germany). Epithelial thickness and lumen area were measured using Leica Application Suite (LAS) microscope software.

#### 4.7.4. Western Blotting Assay

The prostate tissue was homogenized using a homogenizer. Homogenized prostate tissues were lysed using cold radioimmunoprecipitation assay (RIPA) buffer containing a protease inhibitor cocktail. The lysed cell and prostate tissue were centrifuged at 13,000 rpm for 20 min at 4 °C, and the protein concentration was determined using the bicinchoninic acid (BCA) assay. The cell lysates (30 μg protein/sample) were separated by 10% sodium dodecyl sulfate polyacrylamide gel electrophoresis (SDS-PAGE) at 120 V for 90 min and transferred onto nitrocellulose membranes. The membranes were blocked with 5% skim milk at room temperature for 1 h. The membranes were incubated with the various primary antibodies (diluted to 1:1000), including anti-β-actin, anti-SRC-1, anti-PSA, anti-PCNA, anti-EGF, anti-VEGF, anti-TGF-β1 (Santa Cruz Biotechnology, Dallas, TX, USA), anti-AR, anti-Bax, anti-Bcl-2, anti-cyclin D1, anti-p-ERK, anti-ERK, anti-p-p38, anti-p38, anti-p-JNK, anti-JNK (Cell Signaling Technology, Inc., Danvers, MA, USA), anti-5AR2, and anti-IGF-1 (Abcam Inc., Cambridge, MA, USA) overnight at 4 °C. After the membranes were washed, they were incubated with anti-goat immunoglobulin G (IgG) (Santa Cruz Biotechnology, Dallas, TX, USA), anti-mouse IgG, and anti-rabbit IgG (Cell Signaling Technology, Inc., Danvers, MA, USA) conjugated secondary antibodies (diluted to 1:5000) for 2 h at room temperature. We performed immunodetection using an enhanced chemiluminescence (ECL) detection reagent (Advansta Inc., San Jose, CA, USA). Subsequently, membranes were photographed using the Davinch–Chemi Imaging System (Davinch-K., Korea). The chemiluminescent intensities of protein signals were quantified using ImageJ 1.47v software.

#### 4.7.5. Serum Concentrations of DHT

The concentration of DHT in the serum was determined using a DHT ELISA kit (SunLong Biotech Co., Hangzhou, China) according to the manufacturer’s instructions. The absorbance was measured at 450 nm using a spectrophotometer.

### 4.8. Statistical Analysis

The MS datasets processed by MarkerLynx software were subjected to multivariate statistical analysis with SIMCA-P+ version 12.0.1 (Umetrics, Umeå, Sweden). Differences among *Morus* root cultivar groups were visualized by partial least squares discriminant analysis (PLS-DA). The quality of the PLS-DA models was evaluated by the goodness of fit measure (R_2_X and R_2_Y) and predictive ability (Q_2_), and validated by cross-validation with a permutation test (*n* = 200). Based on the variable importance in the projection (VIP) value > 0.7, calculated by PLS-DA, and one-way analysis of variance (ANOVA) with Duncan’s test (*p* < 0.05) using SPSS 17.0 (SPSS Inc., Chicago, IL, USA), the metabolites contributing to the difference among groups were found and identified. The metabolites identified with significant difference (*p* < 0.05) were visualized in a heat map drawn by R with ggplot2 representing the *z*-score transformed data of *Morus* root metabolites. The heat map was plotted in a green–red color scale, with red indicating a decrease and green indicating an increase in the metabolite level.

## 5. Conclusions

In conclusion, the oral administration of *Igsu* (100 mg/kg) decreased the prostate weight in a rat model of TP-induced BPH. *Igsu* also decreased the expression of androgen signaling-related proteins such as AR, 5AR2, SRC1, and PSA, as well as growth factors, phosphorylation of MAPK signaling-related proteins, and apoptosis- or proliferation-related factors in prostate tissues, in addition to reducing the serum DHT levels. However, the molecular mechanisms underlying its effects are yet to be elucidated. To ensure that this substance is safe for use in humans, further research is needed.

## Figures and Tables

**Table 1:** Identification of metabolites contributing to the separation among groups on the PLS-DA scores plots of the dataset analyzed by ESI-positive and ESI-negative modes in UPLC–QTOF MS.

	Peak No.	RT (min) ^a^	Identification	Exact Mass (*m*/*z*)	Fragment Ions (*m*/*z*)	*p*-Value ^b^	VIP ^c^
**Positive [M + H]**	1	3.36	Mulberrofuran G	563.17	453	3.56 × 10^−38^	1.28
	2	3.78	Mulberrofuran I	561.15	451	1.38 × 10^−46^	1.40
	3	4.31	Kuwanon G	693.23	137	5.01 × 10^−22^	1.12
	4	4.69	Kuwanon D	423.18	153	1.88 × 10^−41^	1.12
	5	4.89	Kuwanon H	761.29	205	8.09 × 10^−27^	1.21
	6	5.23	Kuwanon F	423.18	153	2.79 × 10^−28^	0.60
	7	5.44	Luteolin-methyl ester-glycoside fragment	301.07	283, 177, 153	7.22 × 10^−29^	1.35
	8	5.64	Kuwanon A	421.16	365, 153	8.38 × 10^−13^	0.94
	9	5.88	Kuwanon T	423.18	153	3.59 × 10^−31^	0.76
	10	5.96	Morusin	421.16	365	1.92 × 10^−22^	1.11
**Negative [M − H]**	1	3.22	Kuwanon X	581.18	471, 361, 183, 125	1.97 × 10^−19^	0.77
	2	3.66	Mulberrofuran J	579.16	469, 359, 183, 125	1.58 × 10^−27^	1.04
	3	3.84	Kuwanon L	625.17	499, 389, 311, 183, 125	1.14 × 10^−45^	1.03
	4	3.92	Mulberrofuran I	559.13	449, 183, 125	1.09 × 10^−35^	0.84
	5	4.50	Kuwanon K	691.21	581, 539, 419, 379, 353	1.12 × 10^−29^	0.90
	6	4.84	Sanggenon N	421.00	299, 309, 183, 109	3.32 × 10^−42^	1.19
	7	4.97	Kuwanon O	693.00	531, 421, 287, 259, 183, 125	8.52 × 10^−53^	1.04
	8	5.05	Kuwanon H	759.28	581, 539, 379, 353	1.43 × 10^−24^	0.82
	9	5.44	Sanggenol M	761.00	311, 183, 125	6.24 × 10^−49^	1.09
	10	5.54	Kuwanon A	419.14	311, 231, 183, 125	1.01 × 10^−50^	1.11
	11	5.67	Kuwanon E	423.17	297, 125	2.88 × 10^−34^	0.91
	12	5.81	Kuwanon M	839.31	419, 365, 183	3.36 × 10^−34^	0.77
	13	5.91	Mulberrofuran U	647.22	419, 183	2.33 × 10^−26^	0.95
	14	6.05	Kuwanon D	421.16	183, 125	1.37 × 10^−38^	1.28
	15	6.12	Morusin	419.14	297, 217, 191, 173, 109	1.84 × 10^−27^	0.85
	16	6.37	Kuwanon B	419.14	311, 231, 183, 125	1.25 × 10^−35^	1.12
	17	6.50	Kuwanon U	437.19	325, 183, 125	5.50 × 10^−25^	1.20

^a^ RT is the retention time. ^b^ Variable importance in the projection (VIP) values were detected by PLS-DA. ^c^ *p*-Values were analyzed by ANOVA with Duncan’s test.

## Supplementary Materials

The following are available online at https://www.mdpi.com/1422-0067/21/4/1435/s1, Figure S1: Representative ultra-performance liquid chromatography-quadrupole-time-of-flight mass spectrometry (UPLC-Q-TPF MS) profiles of *Morus* root cultivars, Figure S2: Partial least-squares discriminant analysis (PLS-DA) scores, their quality parameters, and heat map for ESI-positive mode (A), (B) and (C) from *Morus* roots with different cultivars, respectively, Figure S3: Box plot graphs of *Morus* roots with different cultivars for ESI-positive mode, Figure S4: Effect of *Morus* roots with different cultivars on the PSA expression in LNCaP, Figure S5: Effect of *Morus* roots according to different cultivars (*Simheung, Daesim, Cheong-il, Sangchon, Daeseong, Suhong, Suwon*, and *Igsu*) on cell viability in LNCaP cells, Figure S6: Western blot analysis of PSA in LNCaP Cells, Figure S7: RT-PCR analysis of mRNA expression VEGF and MMP-2 in LNCaP cells.

## Abbreviations

MR	*Morus* root
TP	testosterone propionate
DHT	dihydrotestosterone
Fi	finasteride
BPH	benign prostatic hyperplasia
5AR2	5-alpha-reductase-type 2
EGF	epidermal growth factor
VEGF	vascular endothelial growth factor
IGF-1	insulin-like growth factor-1
