# Supplementary material for: Metabolomic Analysis of Morus Cultivar Root Extracts and Their Ameliorative Effect on Testosterone-Induced Prostate Enlargement in Sprague-Dawley Rats
Source: Int J Mol Sci. 2020 Feb 20;21(4):1435. doi: 10.3390/ijms21041435 (PMC7073109; doi:10.3390/ijms21041435)
Supplement: Supplementary file 1 [file ijms-21-01435-s001.pdf]

Supplementary Data

Supplementary Figure 1. Representative ultra-performance liquid chromatography-quadrupole-time-of-flight mass spectrometry (UPLC-Q-TPF MS) profiles of *Morus* root cultivars: 1, mulberrofuran G; 2, mulberrofuran I; 3, kuwanon G; 4, kuwanon D/F/T; 5, kuwanon H; 6, kuwanon D/F/T; 7, luteolin-methyl ester-glycoside fragment; 8, kuwanon A/B; 9, kuwanon D/F/T, and 10, morusin analyzed by ESI-positive mode.

Supplementary Figure 2. Partial least-squares discriminant analysis (PLS-DA) scores, their quality parameters, and heat map for ESI-positive mode (A), (B) and (C) from *Morus* roots with different cultivars, respectively. The quality of the PLS-DA socres plots was evaluated by R2X, R2Y, Q2, and p-values (A) and validated by permutation tests (B). The heat map was drawn by R with ggplot2 and the green-red color represents the z-score transformed raw data of *Morus* root metabolites with significant difference among sample groups. Red and green colors indicate a decrease and an increase of metabolite level, respectively.

Supplementary Figure 3. Box plot graphs of *Morus* roots with different cultivars for ESI-positive mode. Box plot graphs show minimum, first quartile, median, third quartile, maximum and outliers.

Supplementary Figure 4. Effect of *Morus* roots with different cultivars on the PSA expression in LNCaP. LNCaP cells were treated with MR cultivars *Igsu* (1, 5, or10 μg/ml) with or without DHT (10 nM), and the mRNA expression of VEGF and MMP-2 was detected by RT-PCR. Data are representative of three independent experiments. Data are expressed as

the means±S.E.M. ** $p < 0.01$ compared with the only DHT treated group.

Supplementary Figure 5. Effect of *Morus* roots according to different cultivars (*Simheung, Daesim, Cheong-il, Sangchon, Daeseong, Suhong, Suwon, and Igsu*) on cell viability in LNCaP cells. Cell viability was measured by the MTT assay. LNCaP cells were treated with MR cultivars (10 μg/ml) for 24 h prior to the MTT assay.

Supplementary Figure 6. Western blot analysis of PSA in LNCaP Cells. LNCaP cells were incubated in medium containing DHT (10 nM) or *Igsu* (1, 5 or 10 μg/ml) for 24 h, and then cell lysates (30 μg) were assayed for expression level of PSA by Western blotting. Data are representative of three independent experiments. Data are expressed as the means±S.E.M. ## $p < 0.01$ compared with the DHT non treated group; ** $p < 0.01$ compared with the only DHT treated group.

Supplementary Figure 7. RT-PCR analysis of mRNA expression VEGF and MMP-2 in LNCaP cells. LNCaP cells were treated with *Igsu* (1, 5, or10 μg/ml) with or without DHT (10 nM), and the mRNA expression of VEGF and MMP-2 was detected by RT-PCR. Data are representative of three independent experiments. Data are expressed as the means±S.E.M. ## $p < 0.01$ compared with the DHT non treated group; ** $p < 0.01$ compared with the only DHT treated group.

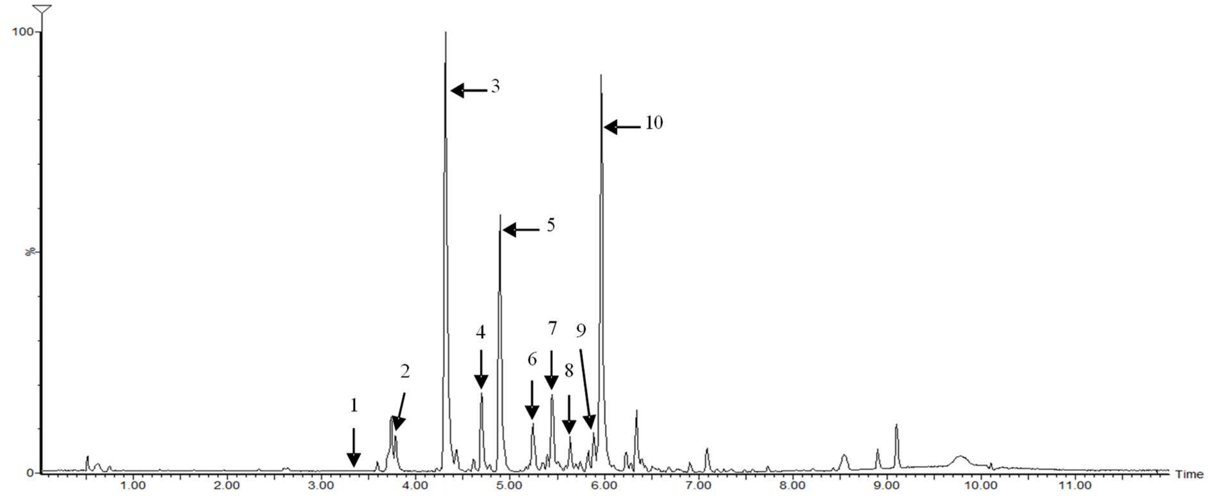

Supplementary Figure 1. Choi et al

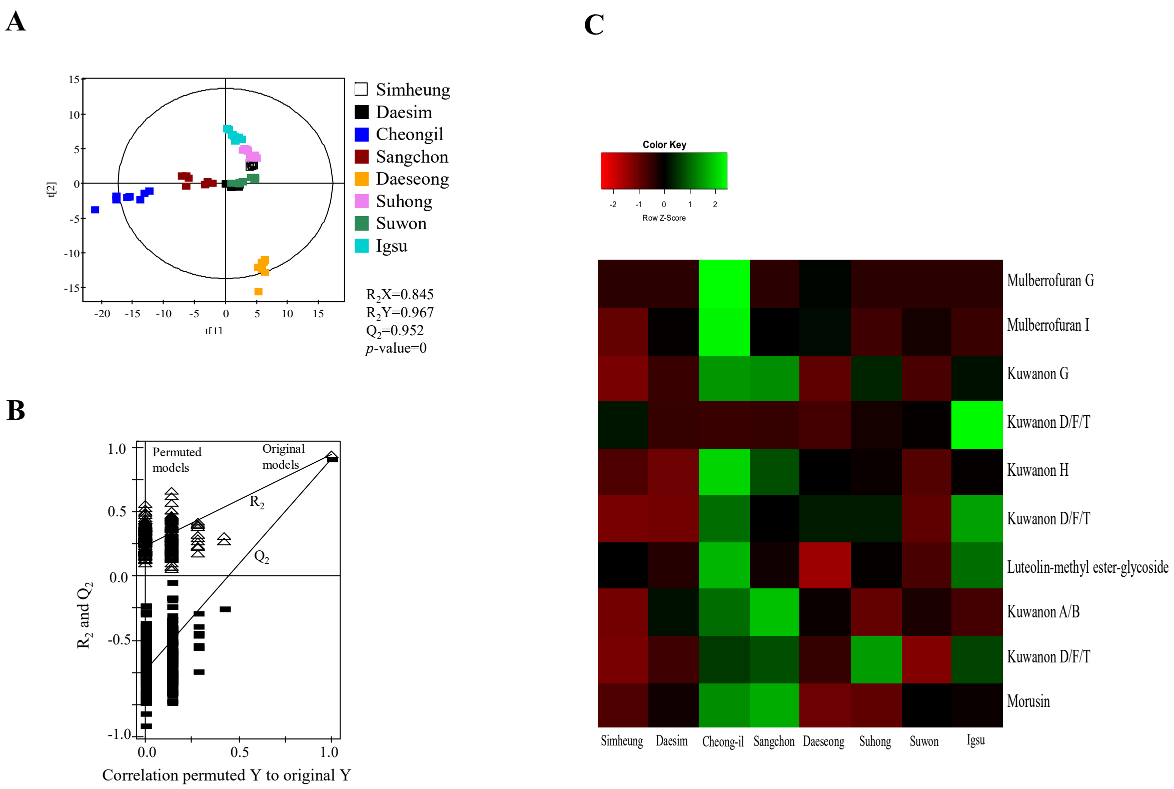

Supplementary Figure 2. Choi et al

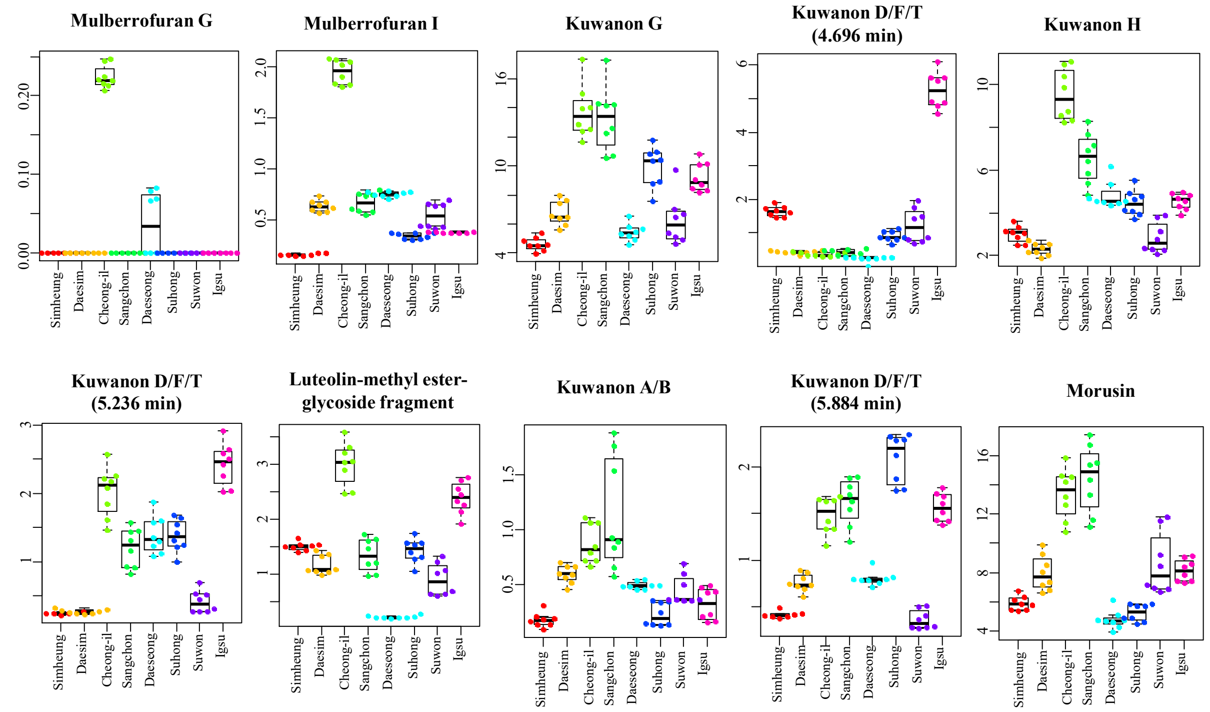

Supplementary Figure 3. Choi et al

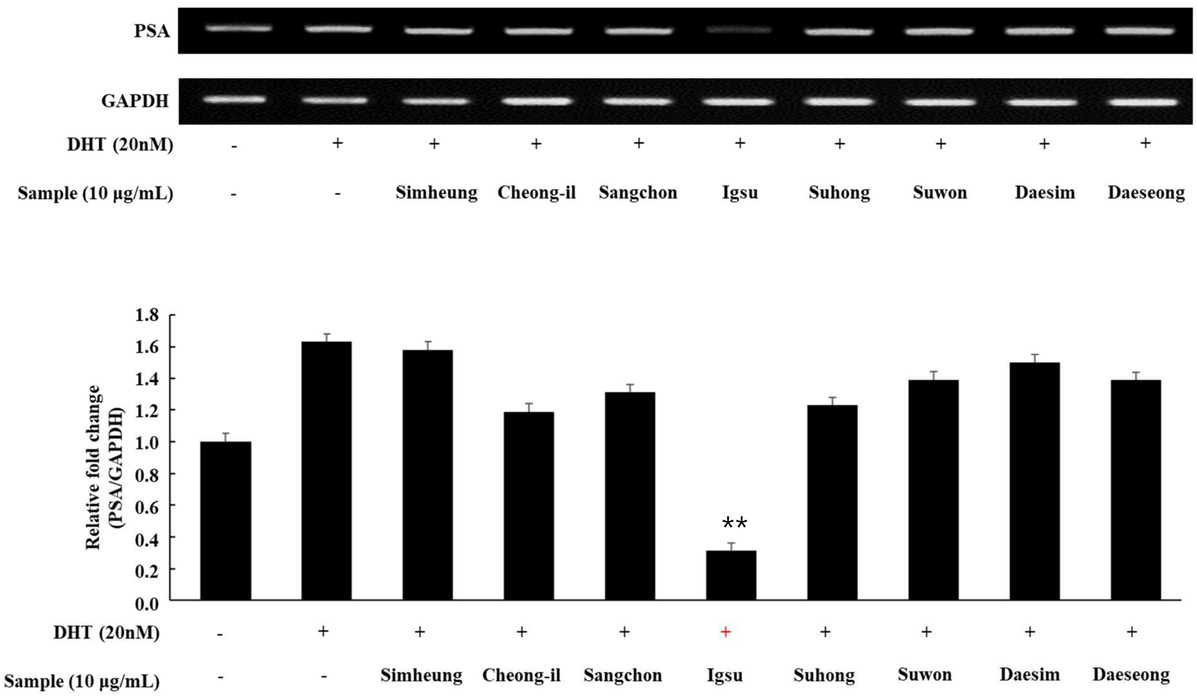

Supplementary Figure 4. Choi et al

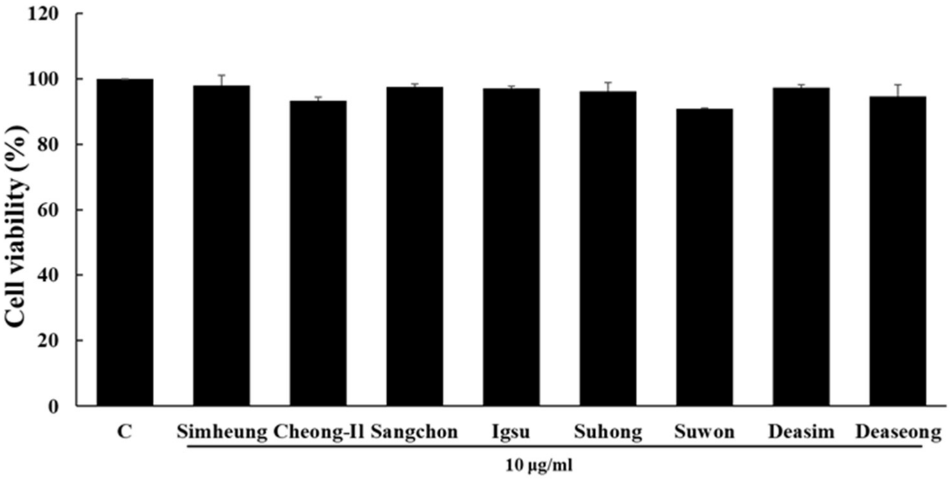

Supplementary Figure 5. Choi et al

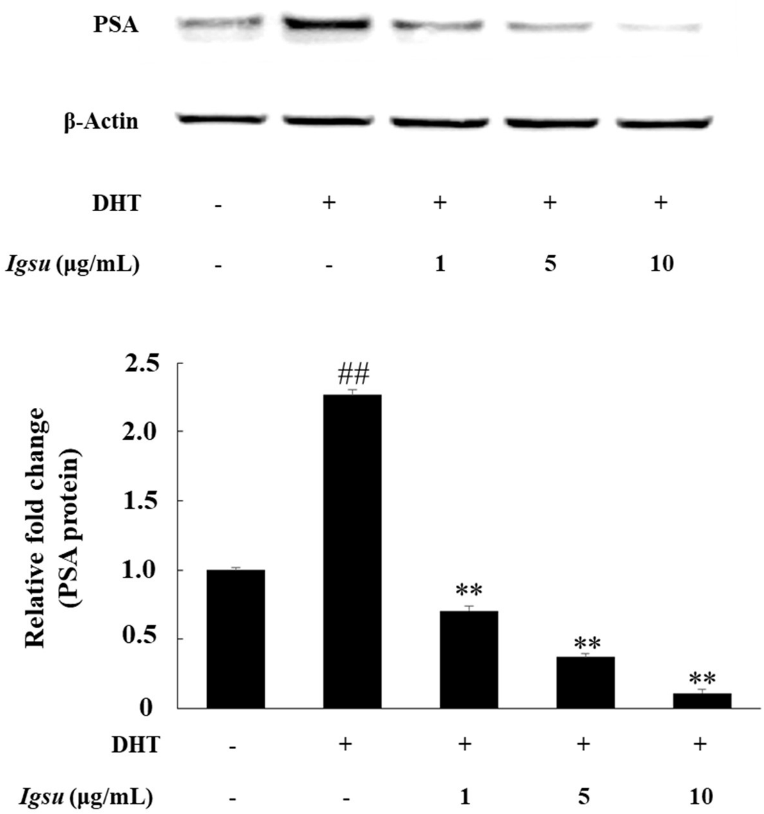

Supplementary Figure 6. Choi et al

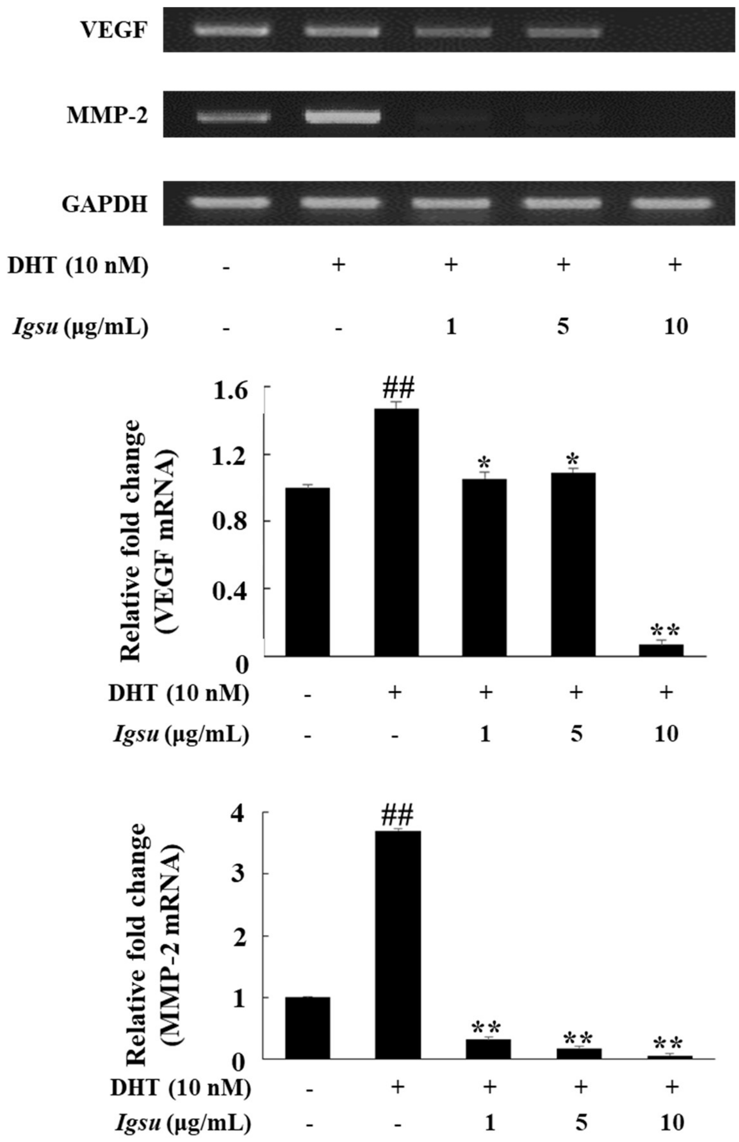

Supplementary Figure 7. Choi et al
